# Intravehicular, Short- and Long-Range Communication Information Fusion for Providing Safe Speed Warnings

**DOI:** 10.3390/s16010131

**Published:** 2016-01-21

**Authors:** Felipe Jiménez, Jose Eugenio Naranjo, Francisco Serradilla, Elisa Pérez, María Jose Hernández, Trinidad Ruiz, José Javier Anaya, Alberto Díaz

**Affiliations:** 1 University Institute for Automobile Research (INSIA), Ctra. Valencia, Km. 7, Madrid 28031, Spain; joseeugenio.naranjo@upm.es (J.E.N.); fserra@eui.upm.es (F.S.); jjanaya_c@gmail.com (J.J.A.); alberto.da@gmail.com (A.D.); 2 Psychology Faculty, Universidad Complutense de Madrid, Campus de Somosaguas, Ctra. de Húmera, s/n, Pozuelo de Alarcón, Madrid 28223, Spain; elisaperez@psi.ucm.es (E.P.); mjhlloreda@gmail.com (M.J.H.); truiz@psi.ucm.es (T.R.)

**Keywords:** speed adaptation, dynamic conditions, information fusion, intravehicular information, wireless communications

## Abstract

Inappropriate speed is a relevant concurrent factor in many traffic accidents. Moreover, in recent years, traffic accidents numbers in Spain have fallen sharply, but this reduction has not been so significant on single carriageway roads. These infrastructures have less equipment than high-capacity roads, therefore measures to reduce accidents on them should be implemented in vehicles. This article describes the development and analysis of the impact on the driver of a warning system for the safe speed on each road section in terms of geometry, the presence of traffic jams, weather conditions, type of vehicle and actual driving conditions. This system is based on an application for smartphones and includes knowledge of the vehicle position via Ground Positioning System (GPS), access to intravehicular information from onboard sensors through the Controller Area Network (CAN) bus, vehicle data entry by the driver, access to roadside information (short-range communications) and access to a centralized server with information about the road in the current and following sections of the route (long-range communications). Using this information, the system calculates the safe speed, recommends the appropriate speed in advance in the following sections and provides warnings to the driver. Finally, data are sent from vehicles to a server to generate new information to disseminate to other users or to supervise drivers’ behaviour. Tests in a driving simulator have been used to define the system warnings and Human Machine Interface (HMI) and final tests have been performed on real roads in order to analyze the effect of the system on driver behavior.

## 1. Introduction

Vehicle speed is a key traffic factor for achieving high levels of traffic flow intensity, but inappropriate speed is a factor in a high percentage of accidents (more than 11% of accidents in Spain in 2012 and over 23% of fatal accidents, according to the database of the Dirección General de Tráfico) [1]. Although statistics show that this concurrent factor has decreased, since in 2008 the above figures were close to 14% and 28%, the data show that this is a problem that must be solved. To adapt speed to traffic, road and environmental conditions are the fundamental premise of the driver. However, drivers can misjudge the geometry and the dangerousness of the road layout by not appreciating all the factors and tend to not consider contingencies that may occur in areas not controlled visually, mainly on rural single-carriageway roads [2].

At present, in addition to the generic and specific limits for certain road sections included in the vertical and horizontal static signals, it is possible to modify the speed limits dynamically via variable information panels. Thus, situations like congestion or a wet road surface can be taken into account to indicate lower limits for that particular stretch. However, particularizing such limits to the type of vehicle remains complex and, even more so, to driving conditions, such as load in the case of commercial vehicles, for example. Thus, in many cases, road signs do not consider the differences in the dynamic behaviour of different vehicle types. Moreover, providing recommended speed limit warnings in rain or fog, for example, must be done as soon as possible once slippery road conditions or poor visibility have been detected. Furthermore, it is quite expensive to implement variable information panels and it is also unaffordable to use them on every road. At the moment, most of them are placed on high capacity roads, but not on single-carriageway roads, where the number of accidents has not decreased in the same way as on other road types. This fact makes it desirable to introduce onboard systems that could play a similar role but at a lower implementation price and affordable for most users.

The desirability of implementing measures to control speed is determined by their effectiveness in limiting the speed, their acceptance by drivers, other users, third parties involved and the overall reduction of the danger and other negative effects. Intelligent speed adaptation (ISA) systems can be classified according to different criteria such as the permissiveness of the system with the driver's actions (informative, voluntary and compulsory types are distinguished), and the frequency of updating speed limits [3,4]. Regarding the second criterion, the systems are classified into fixed, variable and dynamic limits. The first option corresponds to the legal limits of the roads, which ignores many of the features that significantly influence safety (type of vehicle, weather, *etc.*). The variable limits allow a better fit to safety criteria when considering accurate road geometry. However, the consideration of dynamic conditions such as traffic or weather is excluded from these limits. This aspect represented an important technological leap since obtaining that dynamic information in real time involved the incorporation of vehicles communication with other vehicles and/or the infrastructure because onboard information was not enough.

Over recent decades there have been numerous experiences with ISA systems. As examples we can mention those in Sweden [5,6,7], the United Kingdom [8], Denmark [9], France [10], Japan, *etc.* Studies have shown that the greatest potential in reducing accidents is achieved by using dynamic limits and mandatory systems [11]. It has also been found that the acceptance of the mandatory version was much lower than the informative version [12]. However, the analysis performed in [2] showed that most ISA system experiences consider only fixed limits, and only rarely are dynamic limits taken into account because of the technical barriers involved in obtaining and disseminating such type of data.

In Spain, the first experience in this field was a system prototype that suggested variable speed limits [13] which incorporated a detailed digital map of the road geometry and a map of safe speeds according to objective criteria (lateral acceleration, tyre adherence and visibility distance). The system was tested on real roads with positive results in terms of reduction and homogenization of speed [14]. In addition, its potential impact on road safety was analysed [15]. Finally, in order to increase user acceptance, a plan of redefining the criteria and system warnings was developed [16].

However, over the previous developments, speed limits do not take into account changing conditions because external information is needed. In addition, the evaluation of the impact on the driver was developed taking the objective parameters of driving behaviour, but the possible impact on attention was not analysed. These issues are addressed in the present system. Therefore, the main objective sought has been to develop a system that warns the driver in real time of the safe speed in the next road section in terms of geometry, variable conditions and vehicle type. Thus, compared to what has already been developed, this new approach provides innovations related to the fusion of information from different sources:

- Use of the in-vehicle CAN bus to infer possible situations of rain or fog quickly without waiting for wireless communication with external information sources. This fact makes updating dynamic information more flexible because providing information from a control centre involves some delays. Obviously, in-vehicle information should be corroborated with external information in a short time window.
- Use of short-range communications to obtain information from the roadside. This solution could be similar to the implementation of variable information panels on the infrastructure but the cost is much lower and information points on the infrastructure could be placed closer together, providing more continuous information. These communications could be used when local dynamic changes that could not be appreciated by on-board sensors appear (for example, the movement of a shock wave caused by a traffic jam).
- Use of long-range communications to obtain information from a control centre that collects information from different sources (even the vehicles equipped with the system). This information can corroborate data provided from sources 1 and 2, but a certain delay in processing and transmitting is unavoidable. Furthermore, some information, such as the road geometry characteristics of specific road sections, is relocated to the server that is accessed from the vehicles, releasing the onboard device from having this heavy information and easing the updating process.

Finally, the HMI impact has been assessed in a driving simulator and its effect on driver attention is assessed, which goes beyond the mere quantitative effect on the speed profile. The remainder of this paper is organized as follows: Section 2 provides a detailed description of the system architecture. Section 3 and Section 4 explain how information is retrieved by the system in order to estimate the safe speed at every moment. Tests in a driving simulator presented in Section 5 have been used to define the system warnings and HMI. Section 6 shows the final implementation and the impact analysis on driver behavior. Finally, Section 7 presents the main conclusions and highlights the improvements in terms of information and data fusion that this system proposes.

## 2. System Layout

**Figure 1:**
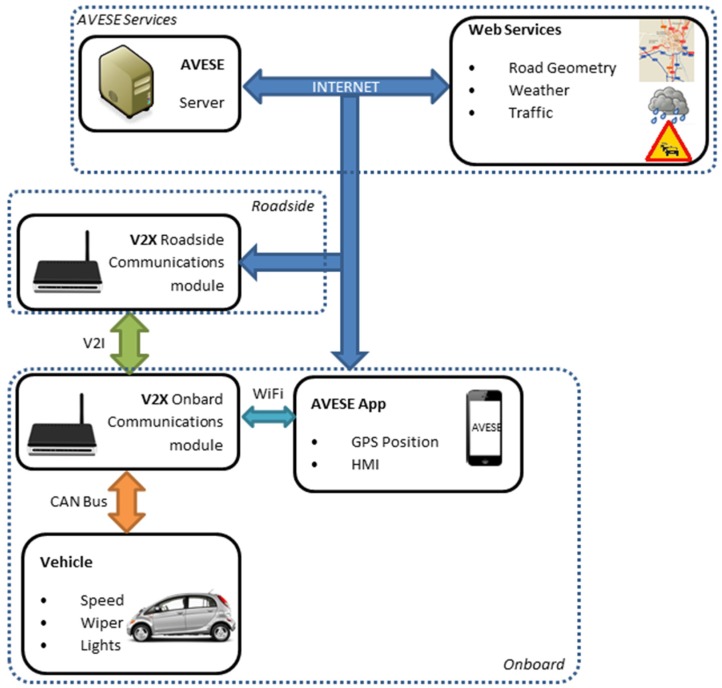
System layout and information sources.

The system structure is based on the initial development described in [13], which includes innovations to provide warnings to the driver in real time while taking into account dynamic conditions. In order to enhance the flexibility of the system, the implementation is performed on a smartphone. This fact is not strictly novel for this kind of system because some other Intelligent Speed Adaptation (ISA) systems rely on the same platform [17,18], but it is the most practical solution for implementation among users of single carriageway roads. The system layout, called AVESE, is shown in Figure 1.

Thus, the system has real-time information on vehicle type, the main factors that can affect its dynamics (load), road geometry in critical sections and road conditions (traffic level and weather conditions obtained from the server or from in-vehicle information systems). With this information, the safe speed is set to the next road stretch by running a mathematical model of vehicle dynamics which includes conditions such as tyre adherence, stopping distance and vehicle lateral acceleration.

In this system, the smartphone GPS receiver is used. Given the expected low-medium positioning accuracy, the map-matching algorithms need to be improved in order to locate the vehicle in the digital map [19]. Moreover, it includes access through wireless communication to the information of the vehicle CAN bus in order to obtain information such as speed, but also of the activation of lights, windscreen wipers, *etc.*, which could be indicators of weather conditions. Similarly, the system has access to a centralized road information server and to roadside communication modules. Information on road geometry at critical sections is stored in the server and dynamic real-time weather and traffic data are also integrated to comprise different information sources. This information is used to calculate the safe speed on each section.

Finally, it should be noted that the communication is bidirectional, so the vehicle sends information such as speed, position, lights activation, and rain detection to the server and the roadside units, in order to generate new updated information that could be used by other road users. On the other hand, the server provides information on the geometry of the road in the following sections and their dynamic conditions to the vehicles equipped with the system, while the roadside units provide much updated weather and traffic condition information.

The method for setting the safe speed is based on the simulation of vehicle dynamics considering different types of vehicles (passenger cars, vans, trucks and buses are taken into account). Using these models, the safety margin is assessed when driving at various speeds in terms of available friction, stopping distance and lateral acceleration [2]. Initially, complex models, both commercial ones such as CarSim and TruckSim [20] and previously *ad-hoc*-developed models [21] are used, although the models need to be simplified to be run in real-time in a smartphone.

Finally, it should be noted that, since vehicle movement information is collected on the server, these data could also be used for monitoring the driver in the event of the server being managed by a vehicle fleet company.

## 3. Server Information

The server information includes data on the road geometry, but the server also receives information about dynamic variables such as weather and traffic conditions. This architecture allows more frequent updates of road data than if this information resides only in the vehicle system controller. Thus, the vehicle sends its position to the server and, if desired, other variables such as speed, wiper and light status, *etc.*, acting as a mobile sensor node [22], while in return the server provides the relevant characteristics of the road section where the vehicle is and the following sections. From these data, the ISA system calculates the safe speed as indicated above.

**Figure 2:**
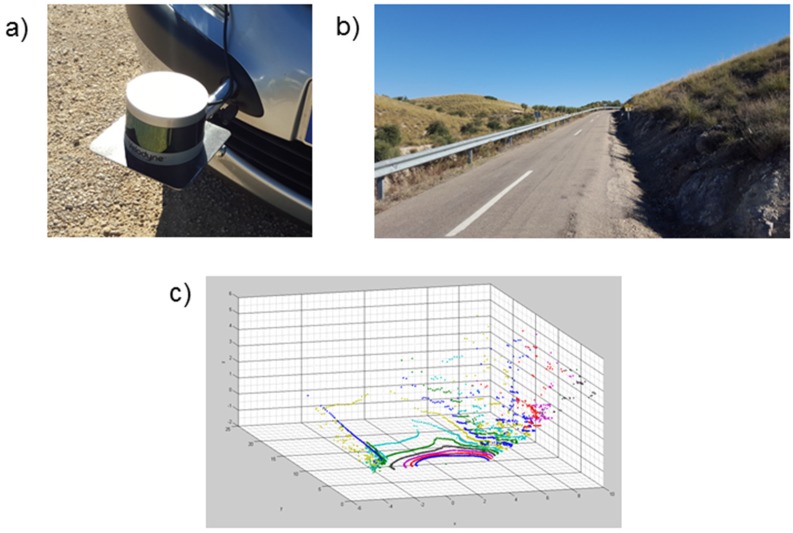
(**a**) Three-dimensional laser scanner; (**b**) Road section example; (**c**) Recorded data of the road surroundings.

The road geometry data that the system needs involves greater detail and accuracy than those required by conventional navigation systems [23]. It is therefore necessary to generate such information, for which the procedure presented in [24] is used, based on a vehicle instrumented with a FES 33 gyroscopic platform inertial system (RMS Dipl.-Ing Schäfer GmbH & Co, KG. Hamburg, Germany), a L-CE non-contact speed sensor (Corrsys-Datron Sensorsysteme GmbH. Wetzlar, Germany) and a G12 GPS receiver (Thales Navigation, Inc. Santa Clara, CA, USA). Additionally, video recording to identify other relevant elements near the road was performed. In this case, we added a three-dimensional VLP-16 laser scanner (Velodyne, Morgan Hill, CA, USA) in front of the instrumented vehicle. Thus, a representation of the road environment was obtained (Figure 2) so risk areas such as narrowing roads or areas of reduced visibility can be identified.

## 4. System Communications

One of the main advantages of the system relies on the multiple sources of information. They could be classified into three groups: on-board vehicle information, roadside units’ information and external server information. All of them are collected by the smartphone, which calculates the safe speed and displays the deceleration information on its screen. For managing these communications the ITS-INSIA V2X communication module was used [25]. This module has been designed following the current standards on V2V and V2I communications, including the IEEE 802.11p protocol in the Physical Layer, IEEE 802.11 in the MAC layer and IEEE 802.2 in the Logical Link Control Layer. The communications module also includes GeoNetworking functionalities, following the ETSI-302-636-4-1 standard and a TCP/basic transport layer based on ETSI 302 636-5-1 to manage the TCP/IP routing in vehicular ad-hoc networks. The modules also include GPS positioning, and a CAN bus interface, thus they can retrieve information from the vehicle to feed the safety services developed. The module also behaves as a WiFi access point, thus a smartphone or any other device can connect to this network in order to retrieve information on the car.

**Figure 3:**
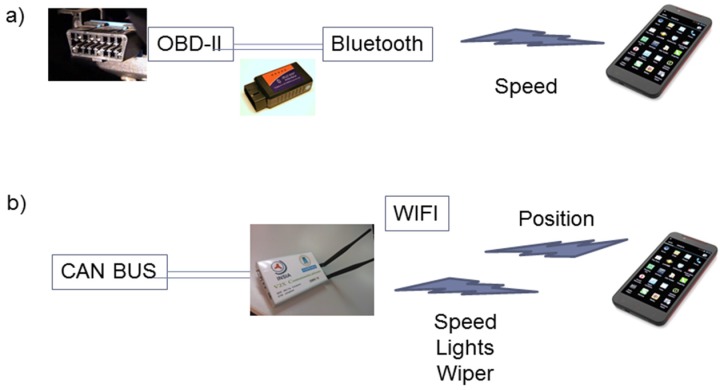
Vehicle information retrieving. (**a**) OBD-II information retrieving; (**b**) CAN-BUS information retrieving.

In the case of onboard information retrieving, two alternatives were tested (Figure 3). In the first one, OBD-II is accessed using Bluetooth directly from the smartphone, but only limited information is available. The second solution involves the use of the specific communications module which makes it possible to obtain the information from the internal CAN bus and send it to the smartphone. More specifically, the information extracted through the CAN bus includes vehicle speed, lights status and wipers status, in order to detect the illumination and rain conditions. The first solution has been tested but the final AVESE prototype has been designed using the second solution.

Furthermore, the module sends this information to the server in two ways depending on the availability of communication systems at the roadside. As priority media, the onboard module will send the vehicle information to the server via V2I communications when roadside units are available in the infrastructure. The V2I roadside units will route the information packages through a dedicated Wide Area Network (WAN) or Internet so that they can be received. In case these units are not available, the module will use the smartphone as router to send the vehicle information to the server via the Internet. The roadside units, when available, can also send dynamic conditions information to the onboard modules.

**Figure 4:**
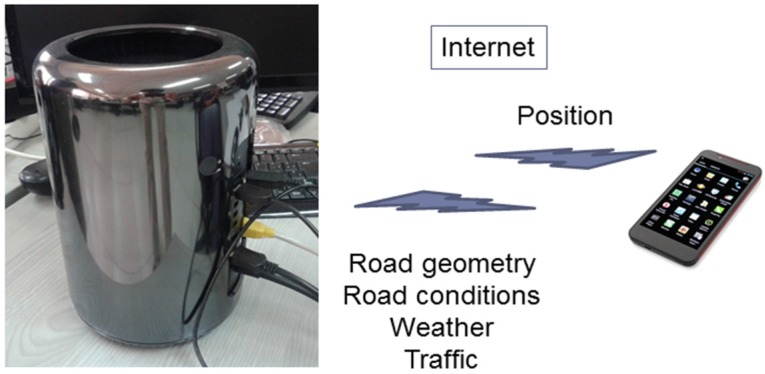
Communication with AVESE server.

Finally, the smartphone is connected via the Internet 3/4 G with the AVESE server in order to retrieve the information on road geometry, speed limits, dynamic conditions and recommendations for each road section (Figure 4). The communication with the server does not have relevant latency requirements so this Internet connection is enough to support the safety system.

## 5. Human-Machine Interface

The user interface has been implemented on the mobile device, optimizing its design on the basis of driving simulator tests with a set of drivers. The interface is not limited to indicate safe speeds but provides warnings in advance to foster better driving. Within this new version of the system, special attention has been paid to the development of the user interface.

### 5.1. HMI Definition

In order to design the HMI, principles to enhance its efficacy have been applied. We can define usability as the perception of a target user of the effectiveness (fit for purpose) and efficiency (work or time required to use) of the interface. This discipline is derived from psychology and ergonomics and is deeply focused to computer science. However, nowadays, the design of HMIs is a process that is based on computer technology, even to be applied to any field. Some standards in the area have been developed, like ISO/TR 16982:2002 [26]. One important part of usability is accessibility; defined as the degree to which a product (e.g., device, service, or environment) is accessible by as many people as possible, including disabled and elderly people. Following these disciplines, we have applied computer science usability concepts to the HMI design of road vehicles in order to develop a set of prototypes that improve safety in road transport.

It is clear that any concurrent task can interfere with the monitoring and processing of the main driver task, which is to safely keep the vehicle on the road and to complete the desired route. Additional cognitive or motor subtasks (lighting a cigarette or speaking on a mobile phone) may cause interferences with the primary driving task. However, some of these interferences are unavoidable, for example, road stimulus. Other interferences like the ones that provide additional on-vehicle information systems can be avoidable and the perturbation caused should be less relevant than the added benefit. Speed or engine speed readouts, GPS navigators, safe headway warnings or passive driving assistances are nowadays considered as indispensable. This is the reason why these secondary stimuli generated by the vehicle must not be removed but adapted in order to cause minimum interference to the driving task. This fact is even more important when new assistance systems that provide extra information are fitted in the vehicle. Consequently, it is critically important to focus two premises in the HMI design: learning capability and efficiency, that is usability. We have defined five fundamental principles to be followed in the design of vehicle HMIs:

(1) Do not disturb. The system and the information content must increase safety. It must avoid producing potentially dangerous behaviours for the drivers or any other road user.
(2) The attention required by the driver when interacting with the HMI must be compatible with the attention demanded by the driving task. Both tasks must be made compatible in order to avoid distractions or any reduction in the driving focus. The necessary attention that will be demanded by the secondary tasks in HMI interacting must be foreseen.
(3) The HMI must not visually disturb the driver. It must be precise. It is important to ensure the minimum distraction of the driver when receiving and using the information provided by the HMI.
(4) The interface must be coherent and compatible. Coherence affects the aspect of the design with elements like colours, icons and sounds that permit a balance between the similarity and differentiation of the presented information.
(5) Visual information must be designed so that the driver can assume the information as quickly as possible and without any negative effects on driving.

Additionally, the information presented must be precise and shown at the right moment to help the driver to correctly face off the situation. These instructions have to be presented to the driver in a way that the driver can evaluate the correct manoeuvre and when it should be performed. In this sense, it is important to determine priorities in the message set shown to the driver. As part of the new development presented in this paper, we proceeded to evaluate alternative user interfaces.

### 5.2. Warning Criteria

The purpose of the driver warning system is not confined to reporting the safe speed on each road section or the following ones, but to provide an orientation on how to adapt the speed safely at every moment. It should be noted that the system is specially designed for road sections with relevant properties that make them have a greater degree of risk than others (curves, intersections, junctions, narrowing, *etc.*). Thus, “normal sections” (Zone A) and “potentially risky sections” (Zone B) are distinguished, and warnings are provided when approaching a Zone B section or inside them.

Warning criteria are different depending on the area where the vehicle is. Thus, the system presented in [14] provides the following messages: (1) speed over legal limit indicator; (2) nine indicators that are activated by increasing the deceleration required to adapt the speed to a safe limit in the following Zone B (while the vehicle is in a Zone A) or that all of them are activated if the safe speed is exceeded in a Zone B. However, this solution involved a practical problem for drivers when the difference between the actual speed and the safe one is small in both Zones A and B, since the interface provides a message that may be more alarming than necessary. This forced to introduce the concept of variable reaction time, which decreases as more deceleration warnings are activated. Therefore, the deceleration that marked the activation of the 9 indicators can be calculated by Equation (1):

(1) $$a=\frac{v^{2}-{v_{s}}^{2}}{2\cdot \left(d-tr\cdot v\right)}$$

where *v* is the vehicle speed, vs is the safe speed in the next Zone B road section, *d* is the distance between the vehicle’s actual position and the beginning of Zone B and *tr* is the reaction time.

**Figure 5:**
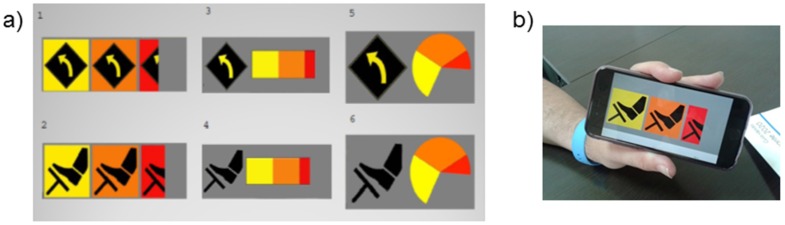
(**a**) Different interface designs; (**b**) Implementation of the interface in a smartphone.

This solution has not been misperceived by users [16], but drivers considered it was not easy to learn. Therefore, we proceeded to make changes. Firstly, the indicator showing the legal speed has been exceeded was removed, because it was perceived as not useful in the preliminary tests on single carriageway roads. Moreover, warnings of varying intensity have been maintained when approaching type B areas but with a continuous evolution. Different formats will be evaluated later (Figure 5).

Finally, the criteria have been reformulated, so variables such as the difference between the actual speed and the safe one and the necessary deceleration *a* are considered jointly. Equations (2)–(4) show the criteria adopted:

(2) $$speed\;component=\{\begin{array}{ll} 0 & if\;v<v_{s}\\ 100\frac{v-v_{s}}{v_{s}} & if\;v_{s}\leq v\leq 1.5\cdot v_{s}\\ 50 & if\;v>1.5\cdot v_{s} \end{array}$$

(3) $$deceleration\;component=\{\begin{array}{ll} 50\frac{a}{a_{\text{max}}} & if\;a\leq a_{\text{max}}\\ 50 & if\;a>a_{\text{max}} \end{array}$$

(4) $$warning\left(\%\right)=\{\begin{array}{c} speed\;component+deceleration\;component\\ 2\cdot speed\;component \end{array}\;\begin{array}{c} in\;Zone\text{​ }A\\ in\;Zone\;B \end{array}$$

where *v_s_* is the safe speed, *a* is the deceleration calculated by Equation (1) using a constant reaction time when driving in Zone A and *a_max_* is the maximum deceleration in safe conditions.

### 5.3. HMI Assessment

The aim of the study is to evaluate different interfaces to inform the driver of the safe speed. The analysis will allow selecting the interface that best meets the criteria for implementation in a mobile phone. For the design, the recommendation of the European Commission of 26 May 2008 concerning information and communication systems on safe and efficient vehicles was taken into account. Twenty participants took part in this study (10 men and 10 women) aged between 20 and 69 years. The main characteristics of the sample are presented in Table 1.

**Table 1:** Drivers involved in driving simulator tests.

	Average Value	Standard Deviation
Age (years)	35.35	13.23
License experience (years)	13,35	11.62
Hours driven weekly (h)	7.48	4.34

**Figure 6:**
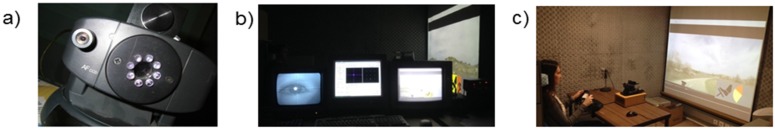
Laboratory tests. (**a**) ASL Model 504 Ocular system log; (**b**) Control computers for running simulator and eye-tracking system; (**c**) Driver performing a simulation.

Tests were conducted on a driving simulator, including steering wheel and pedals. In order to analyze the driver attention paid to the system, an eye tracking system was used. The Model 504 Ocular system log (ASL, Billerica, MA, USA) is a remote unobtrusive eye tracking system designed to measure the diameter of the pupil and coordinate where the user is looking. The eye movement camera launches an infrared beam. Through the reflection of the pupil and cornea, it determines where the subject is looking. The vertical and horizontal position of the eye, pupil diameter and 16-bit external data were recorded at a frequency of 50 Hz. Along with these eye movement data, the system returns an image of the subject’s field of view and their glance superimposed by a cursor in real time. The system has an accuracy of 0.5° of visual angle. Figure 6 shows the laboratory tests performed.

A total of six simulated scenarios of 5 minutes each were used with different interfaces. The presentation order was counterbalanced. After each scenario, the participants answered an Acceptance Scale of education systems [27]; the SUS Usability Scale [28]; and the Rating Scale of Mental Effort [29]. At the end, they also answered the I-Driving Scale [30] and questions on demographics such as the loss of driving points and motives, the number and type of accidents. The interfaces presented in Figure 5 were tested. The dependent variables considered are grouped into different categories:

- Behaviour (number and intensity of braking)
- Distraction (flicker number, number and duration of eye fixations)
- Acceptance of the system (satisfaction, utility and usability)
- Mental load (using a subjective measure, RSME, and a physiological measure, pupil dilation)

**Figure 7:**
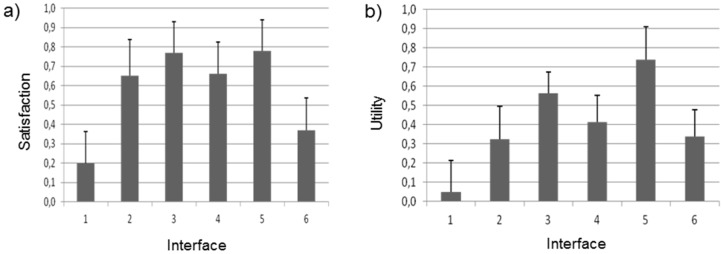
Average value in (**a**) Satisfaction and (**b**) Utility.

For each dependent variable, univariate ANOVA repeated measures were performed. Significant differences were found between the interfaces in the category of Satisfaction, interfaces numbers 2, 3, 4 and 5 being considered equally satisfactory, and less satisfactory 1 and 6 (F(5; 95) = 2.69, *p* = 0.026). For the Utility variable, significant statistical differences are found (F(5; 95) = 3.17, *p* = 0.011). Now, interfaces 3 and 5 are considered more useful than 1, but equally useful to 2, 4 and 6. Regarding usability, there were no statistically significant differences among the six interfaces (F(5; 95) = 1.93, *p* = 0.096). Figure 7 illustrates the differences between interfaces in satisfaction and utility.

**Figure 8:**
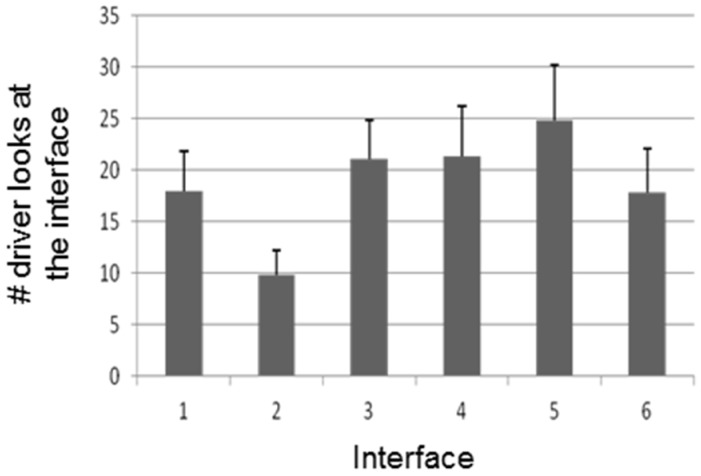
Number of times the user looks at the interface.

Furthermore, the number of times the user looks at the interface is different considering the interface type (F(5; 95) = 3.96, *p* = 0.003). More specifically, interface 2 is the one that requires least attention (Figure 8). On the other hand, there were no significant differences in eye fixation durations (F(5; 95) = 0.81, *p* = 0.55) and number (F(5; 50) = 1.11, *p* = 0.37) and intensity of braking processes (F(5; 65) = 1.84, *p* = 0.12).

Using the RSME scale regarding the mental workload, no statistical differences were found between the analysed interfaces (F(5; 95) = 1.77, *p* = 0.13). The same conclusions can be found when studying pupil diameter (F(5; 95) = 0.9, *p* = 0.48) and flicker number (F(5; 95) = 0.89, *p* = 0.49). For this reason, it was concluded that all the interfaces produce similar levels of mental workload. On the other hand, a significant difference was found between braking intensity and mental workload (R = −0.538, *p* < 0.05). Results show that the greater the mental workload, the lower the reaction of the driver to the warnings (lower braking intensity).

To conclude, the results show that there are no significant differences between interfaces in most of the variables (number and intensity of braking processes, flicker frequency, pupil diameter, eye fixation duration, *etc.*). But, in Variable Satisfaction, interfaces 1 and 6 present the worst results and interface 1 is the one that drivers consider to be the least useful. In this regard, more than one option could be a proper selection for providing safe speed warnings to the driver. However, it has been corroborated that interface 2 causes less distraction from the primary task of driving and this is one of the key criteria that should be taken into account in an HMI design.

Finally, findings should be mentioned including, for example, in [31] which highlight that driver simulator results cannot be directly extrapolated to real roads. However, although the test conditions could affect driver behaviour, we considered that it is not a situation that would invalidate our results, since the purpose of the study focuses on the acceptance and driver response to each interface type, and not so much on their response during the driving tasks.

## 6. Final Implementation

**Figure 9:**
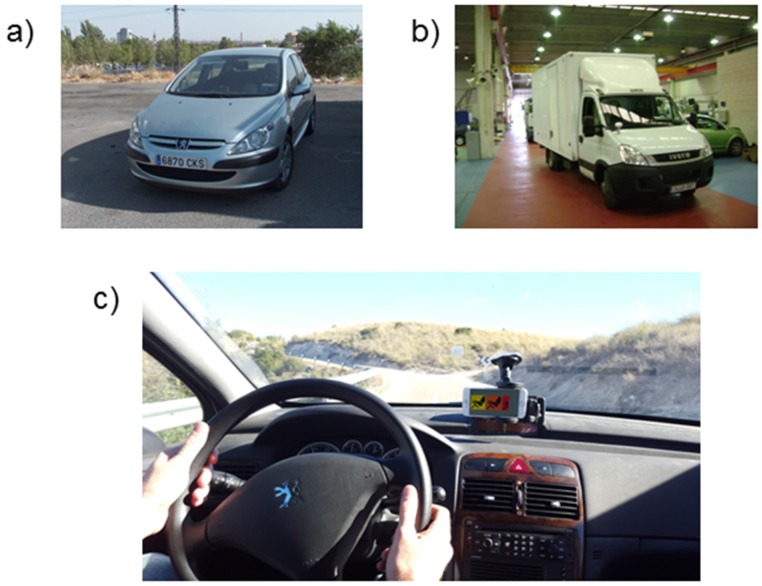
Implementation of the system in 2 vehicle types. (**a**) Passenger car; (**b**) Van; (**c**) System implementation.

The ISA system has been implemented. Considering the above analysis, interface 2 was selected for the implementation in the final system. It can be used in any kind of vehicle. Figure 9 shows the use of the system in two different types of vehicles: a passenger car and a van, whose load (variable that plays an important role on vehicle dynamics) could be substantially changed.

References [14,16] present the results of the impact assessment of the original system on driver behaviour on single carriageway roads, for which the system could be most useful. The vehicle was instrumented and the collected information was focused on quantitative speed profile information and actions of the driver on vehicle control, such as the speed profile followed by each driver, and then the information was compared with safe speed limits, travel time and average and maximum speeds, exceeding the legal speed, exceeding the safe and legal speeds and system messages monitoring. The study of the influence of the system on driving behaviour was focused mainly on most critical road sections where the system gives advance warning of the appropriate speed and how the speed during approach should be modified. There was no significant evidence that the warning system reduces speeding above the legal limit in areas of generic limitation. But it does produce a better fit of the average speed in areas of specific limits. In addition, the system causes greater homogenization of speeds, so that drivers generally do not exhibit significant variations in speed along the road singularity, and there are no significant discrepancies among different users in their speeds, which are in a relatively narrow range close to the safe limit below the usual range in the absence of the system. Both average speed reduction and traffic homogenization are two aspects that are successful in improving traffic safety without any significant travel time increments [32,33].

Trials of the new system have been conducted in the same conditions as the above. The test site was the M-315 road in the surroundings of Madrid (Spain). The road selection was made in order to find a route in which the real effectiveness of the system would be valuable. This is a single carriageway road with several sharp bends with low visibility stretches. Figure 2 shows an example of the appearance of one of this road sections. These characteristics make that the driver finds difficulties in assessing the most convenient and safe speed at every moment. Furthermore, only generic road speed limits are provided, but little information is provided on specific dangerous curves or crossings. In conclusion, in this kind of road, the system would complement the driver perception and the signals.

Ten drivers were involved in the tests. The results have been very similar in terms of driver behaviour, detecting very similar effects as shown in Table 2. Speed reduction and homogenization in Zone B sections have been detected for every driver. In this regard, the differences among drivers are reduced in Zones B (from 18.22 km/h to 11.78 km/h) and maximum and median speeds are reduced in order to fit them to the safe limit established by the system (from 6.53 km/h to 2.50 km/h and from −0.28 km/h to −2.96 km/h, respectively). The explanation for this effect is quite clear: the system provides homogeneous information of the danger degree of the road sections so all drivers try to adapt their speed to the same limit. Furthermore, the system leads to more constant driving speeds in Zone B road sections (the difference between maximum and minimum speeds has fallen in average value from 13.54 km/h to 1.45 km/h), so drivers adapt their speed before these stretches begin and avoid braking inside them. In comparison with other previous speed adaptation systems, this one provides warnings that encourage to reduce speed in advance providing specific information of how to brake, and the braking starting point and the generation of new warnings for a more severe braking manoeuvre are defined depending on the current speed and the target speed in the critical road section. Other ISA systems only provide the speed limit in the following singular stretch but the driver decides when and how to adapt the speed. However, travel time (or average speed) is not significantly influenced by the system because it only acts on Zone B sections and their impact on total travel time is quite small (differences below 1% in average value). This fact could be an advantage for driver long-term acceptance because they could appreciate that driving safely with the system does not influence negatively on travel time. Of course, it would be advisable to analyse whether the travel time perception corresponds with actual data in a long-term use scenario, because warnings suggesting decelerations could produce some confusion in the drivers’ subjective perception. Finally, it should be noted that, although the long-term acceptance was not measured because the tests conditions were not adequate for this purpose, the qualitative acceptance expressed by drivers after testing the system has increased from previous tests in which other interfaces or warnings were used [2,14,16].

**Table 2:** Test results on M-315 single-carriageway road (Madrid, Spain) (*N* = 10).

	**Average Value**	**Standard Deviation**		
Differences of average travel speed with and without the system (%)	−0.58	0.81		
Differences of travel time with and without the system (%)	0.81	0.79		
	**Without the System**		**With the System**	
	**Average Value**	**Standard Deviation**	**Average Value**	**Standard Deviation**
Frequency of safe speed exceeding (%)	18.25	-	9.625	-
Difference between maximum and safe speeds in Zones B (km/h)	6.53	8.36	2.50	5.95
Difference between median and safe speeds in Zones B (km/h)	−0.28	6.66	−2.96	5.14
Difference between maximum and minimum speeds in Zones B (km/h)	13.54	7.23	10.45	5.31
Maximum speed differences among drivers in Zones B (km/h)	18.22	3.56	11.78	5.83

## 7. Conclusions

In this paper a driving assistance system implemented in a mobile phone has been presented. The system can be used for any type of vehicle (passenger car, van or bus) and has configurable parameters that allow it to provide more reliable estimations of the safe speed and recommendations for its adaptation.

One of the great advantages of the proposed system is the ease of implementation in the vehicle so that any user with minimal cost and their own mobile phone could use it by downloading an App. Furthermore, the open system design, based on an external information server makes the system much more flexible to incorporate new information as it can be generated by other data sources. Then, the server can retrieve information from other dynamic databases, including changes to road geometry provided by road administrators (with the possibility of containing a very detailed description), weather conditions and weather forecasts provided by meteorological agencies, or traffic conditions using cameras mounted on the infrastructure, sensors on the road surface, sensors on the roadside or floating vehicles.

Regarding the socio-economic impact, the system can contribute to a reduction in the speed and, above all, a reduction in the variability of this speed, which has been proved to be a major cause of traffic accidents and incidents. Furthermore, due to the layout of the system, it is expected that the main speed reductions and reductions of its variability will be produced on critical road sections and on single-carriageway roads where safety equipment is less than on high capacity roads. Thus, it is expected that the system could contribute significantly to the objectives of Spanish Road Safety in reducing traffic fatalities by 50% in the period between 2011 and 2020 [34].

Also, it should be kept in mind that the ISA system is informative (non-mandatory), which favours its acceptance [12]. Moreover, in the new system, in line with the conclusions of [35] where drivers showed greater willingness to accept assistance systems in adverse conditions, the adaptation of safe speed limits based on changing conditions allows the estimated safe speeds to better meet user expectations, although this has not been corroborated experimentally because of the small size of the sample of drivers involved in the trials. In the future, tests with a larger sample of drivers will be performed and long-term acceptance should be assessed, as well as the final effect of the system in the drivers’ way of driving, because some little corrections are expected from the first results when the drivers get used to the system.

To conclude, the system represents a technological advance over today’s driving assistance systems, overcoming the limitations of supervision and control of current speed adaptation systems, based only on information from the vehicle itself, because information from the outside is collected via wireless communications.
